# Recent Advances in Micro-/Nanoplastic (MNPs) Removal by Microalgae and Possible Integrated Routes of Energy Recovery

**DOI:** 10.3390/microorganisms10122400

**Published:** 2022-12-03

**Authors:** Abdelfatah Abomohra, Dieter Hanelt

**Affiliations:** Aquatic Ecophysiology and Phycology, Institute of Plant Science and Microbiology, University of Hamburg, 22609 Hamburg, Germany

**Keywords:** biofuel, plastic waste, bioremediation, marine environment, green energy

## Abstract

Reliance on plastic has resulted in the widespread occurrence of micro-/nanoplastics (MNPs) in aquatic ecosystems, threatening the food web and whole ecosystem functions. There is a tight interaction between MNPs and microalgae, as dominant living organisms and fundamental constituents at the base of the aquatic food web. Therefore, it is crucial to better understand the mechanisms underlying the interactions between plastic particles and microalgae, as well as the role of microalgae in removing MNPs from aquatic ecosystems. In addition, finding a suitable route for further utilization of MNP-contaminated algal biomass is of great importance. The present review article provides an interdisciplinary approach to elucidate microalgae–MNP interactions and subsequent impacts on microalgal physiology. The degradation of plastic in the environment and differences between micro- and nanoplastics are discussed. The possible toxic effects of MNPs on microalgal growth, photosynthetic activity, and morphology, due to physical or chemical interactions, are evaluated. In addition, the potential role of MNPs in microalgae cultivation and/or harvesting, together with further safe routes for biomass utilization in biofuel production, are suggested. Overall, the current article represents a state-of-the-art overview of MNP generation and the consequences of their accumulation in the environment, providing new insights into microalgae integrated routes of plastic removal and bioenergy production.

## 1. Introduction

Currently, plastic is an integral part of human life, and its utilization cannot be avoided in one way or another. It is one of the most renowned synthetic materials and is invading the natural ecosystem with many negative consequences. It is widely used in different industries, including manufacturing, aerospace, construction, cosmetics, and packaging. Due to its comparatively low production costs and unique physicochemical characteristics, the annual production of plastic has increased dramatically in recent years. Annual global plastic production increased from 2 Mt in 1950 to 380 Mt in 2015, which was accompanied by the generation of 6300 Mt of waste plastic [1]. In 2016 alone, global waste plastic generation was estimated at 242 Mt, representing 12% of all solid wastes [2]. The majority of waste plastic produced accumulates in the environment, while only 9% is currently recycled [3]. Therefore, it is predicted that oceans will have more mass accumulation of plastic waste, making exploring innovative technologies for waste plastic recycling and safe disposal a tough challenge all over the world. According to the European Plastics Manufacturers database [4], high- and low-density polyethylene (HDPE and LDPE, respectively), polypropylene (PP), and polyvinyl chloride (PVC) are the dominant plastic forms, representing together about 59% of the total amount of plastic produced globally. However, the remaining 41% includes polystyrene (PS, 6.7%), polyethylene terephthalate (PET, 7.4%), polyurethane (PUR, 7.5%), and other polymers, such as polybutylene terephthalate (PBT), acrylonitrile butadiene styrene (ABS), polymethylmethacrylate (PMMA), and polycarbonate (PC).

**Figure 1:**
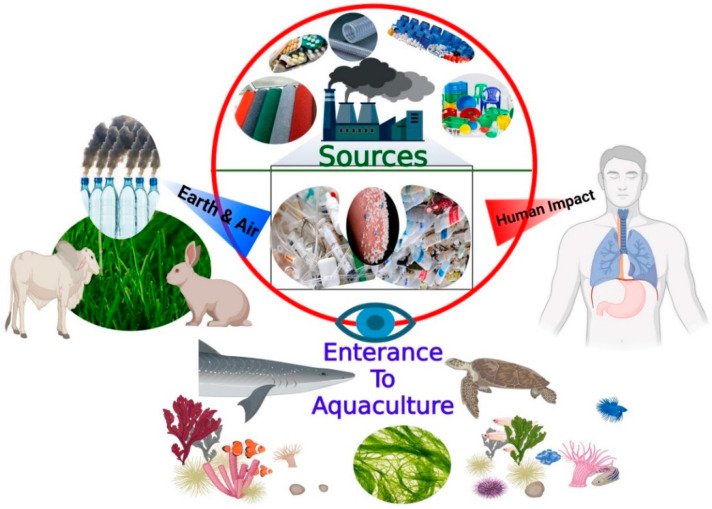
A schematic diagram showing the main sources, entrance, and widespreadness of MNPs in the environment, extending to humans.

Plastic particles have been detected in all environmental ecosystems, including marine surface water and the seabed, surface freshwater and sediment, soil, and even groundwater, as well as in the atmosphere (Figure 1) [5,6,7,8]. Plastic particles undergo weathering and fragmentation by various natural forces once released into the environment. Such natural forces include ultraviolet (UV) radiation, mechanical forces of water, and biological degradation, resulting in the formation of microplastics (MPs) and nanoplastics (NPs) [8]. “MP” is a term used to refer to any synthetic solid plastic polymer with a diameter of ≤0.5 mm [9] generated by either primary or secondary processes [10,11]. Although there is no established definition for “NPs”, usually the term refers to particles with similar origins and compositions to MPs that have smaller sizes of ≤100 nm—much smaller than the algal cell diameter [12,13,14]. MPs and NPs (MNPs) may enter the environment directly through domestic discharges and industrial effluents from cosmetics, cleaning products, and synthetic fibers [15], finding their way eventually into the human body (Figure 1), resulting in negative impacts throughout the whole ecosystem. Despite the reported efficiency of microorganisms in harboring keys for the circular bioeconomy that could help fight plastic pollution and rising CO_2_ levels [16], the potential of microalgae to mitigate the risk posed by MNPs within the circular bioeconomy framework is a subject that requires further study. Therefore, the present work is a timely review article which aims to highlight the risk of MNPs to humans as well as to the environment. In addition, different technologies used for the mitigation of MNP risks are discussed. The interaction of MNPs with microalgal cells and the consequent impacts are presented. In addition, possible routes of dual use of microalgae in MNP removal and biofuel production are suggested.

## 2. Distribution and Implications of MNPs

Due to their small size and wide distribution, studying and tracking MNPs is intrinsically challenging, making MNPs more difficult to control. Specifically, different routes by which MNPs can reach different platforms, the timeframes needed to determine their breakdown durations in order to provide reliable measurements, and future prospections/evaluations are extremely challenging [17]. However, it is estimated that 0.8–2.5 Mt of MNPs are ending up in oceans, of which 44,000–300,000 tons and 63,000–430,000 tons of MPs reach agroecosystems annually in Europe and North America, respectively [11,18]. Another estimation predicts that 5 trillion plastic particles are floating in oceans, with a total mass exceeding 250,000 tons [19]. Compared to all other wastes, plastic accounts for about 60–80% of anthropogenic litter in both terrestrial and aquatic environments [20]. As mentioned in the previous section, MNPs may derive from primary or secondary sources. The first include MNPs in medicinal products, textiles, and personal care products [21,22], as well as the initial manufactured plastic pellets [23]. The second include MNPs produced from the breakdown of larger waste plastic items, including plastic nets, containers, films, line fibers, and tires [24]. Primary MNPs are usually released into industrial and domestic wastewater, which finally enter estuaries and rivers [25]. However, secondary MNPs are much larger sources of plastic pollution in marine systems, with expectations that their abundance will increase enormously due to the unceasing discharge of waste plastic from a wide variety of origins [26]. In addition, the tiny size of MNPs increases their specific surface area, which enhances their bioavailability to aquatic organisms, threatening all living organisms due to the spread of these particles throughout the food chain [26].

As shown in Table 1, MPs have been extensively detected globally in marine ecosystems [27,28]. For instance, extensive pollution by MPs was recorded in surface waters of the northwestern Pacific Ocean, at concentrations ranging from 640 to 42,000 items/km^2^, based on current action [28]. Owing to its receiving huge amounts of waste plastic from the surrounding urban areas, MPs were abundantly recorded in the semi-enclosed Mediterranean Sea, also [26]. MNP distribution is much higher in water bodies close to urbanized areas than in those close to rural areas because of the differing rates of anthropogenic activities. For instance, a previous study evaluated MP distributions in different lakes at the very center of Wuhan city, China, which are surrounded by densely populated residential areas [29]. The results showed the highest MP concentrations in the surface waters of Huanzi Lake (8550 items/m^3^) and Bei Lake (8925 items/m^3^), due to high anthropogenic activities in those areas. After traveling over long routes in marine environments, sediments are the final destinations of MNPs, and high amounts can be detected in marine sediments. In this context, a recent study reported 142 and 155 items/kg dw as the average MP concentrations in offshore sediments from the East China Sea and the Yellow Sea, respectively (Table 1) [30]. It can be noted from the table that water, sediment, and wastewater all contain MNP particles, their levels significantly varying depending on location. In addition, sediment depth and water flow rate, as well as distance from the shoreline, significantly influence MNP concentrations in marine sediments. The abundance of MNPs near the shoreline (210–240 items/kg dw) was reported to be much higher than in deep water (60–90 items/kg dw) [30].

Other potential sources of MNPs are wastewater treatment plants (WWTPs), the presence of MNPs in WWTP effluents having been confirmed by many previous studies. For instance, Mintenig et al. [31] examined the MPs in treated water compared to raw water at a drinking water treatment plant, where MPs were detected in 10 out of 24 studied water samples, with average contents of 0.7 particles/m^3^ in the treated water compared to 7 particles/m^3^ in the raw water. Concerns associated with the negative impacts of MPs are attributed to many issues, including not only the direct toxicity of plastics to biota but also their activity as vectors of other pollutants and invasive organisms/pathogens, as well as the toxicity of adsorbed additives or those used in plastic production [12]. In this context, particles of MPs can adsorb many toxic compounds from the surrounding environment, such as heavy metals (e.g., Cu, Ni, Pb, and Zn) and persistent organic pollutants (POPs) (e.g., polychlorinated biphenyls (PCBs), polycyclic aromatic hydrocarbons (PAHs), polybrominated diphenyl ethers (PBDEs), and dichlorodiphenyltrichloroethane (DDT)) [12,32]. In addition, chemicals added during the plastic manufacturing process might exist with plastic particles, such as bisphenol A (BPA) and phthalates [33,34]. Previous studies have reported that non-polymeric additives can be used as preferred carbon sources for microorganisms due to possible biodegradability over time [35,36]. However, the possibility of these contaminants being transferred into edible seafood poses a significant concern regarding food safety and quality [37].

Compared with MPs, NPs are less well-explored, while the downsizing of waste plastic from micro- to nanoscale was reported to be accompanied by significant changes in physicochemical properties [8]. In addition, fewer studies on NPs have focused on the toxic effects of NPs on marine organisms in the marine ecosystem, including fish, bacteria, and algae [15,38,39]. Aquatic organisms can easily ingest NPs, which ultimately reach various organs and accumulate in the aquatic food chain, including phytoplankton, zooplankton, fish, crustaceans, snails, and marine mammals [40]. The exact translocation mechanisms of NPs are still to be explored, but plastic particles were confirmed to be transported from the gills and/or digestive tracts to the circulatory systems of these organisms [40]. The impact of 51 nm polystyrene NPs on fish through transfer along the food chain from producers to final consumers, as well as direct waterborne exposure, was evaluated [41]. The results showed that NPs induced liver histopathological changes in the fish, which were attributed to direct exposure to NPs, and had the ability to be transferred to fish along the food chain. The latter aspect establishes a confirmed route for MNPs spreading throughout the food chain to reach humans as well [42,43]. The spread has been confirmed by various different types of MNPs detected in human feces [44], confirming the ingestion of MNPs from a variety of sources and their ability to be excreted via the gastrointestinal tract. Particles of MNPs were also detected in human colectomy specimens [45], providing proof that these particles can reach the human colon. MNPs were also detected in the blood [46] and placentas of pregnant women [47,48], which raises a serious concern about the impact on subsequent generations. Therefore, MNP pollution represents a great concern as a potential threat to human health and the whole ecosystem.

**Table 1:** Distribution and concentrations of microplastics in marine environments (water bodies and sediments) as well as wastewater effluents in different regions of the world.

Region/Country	Location	Source	Amount/Concentration	References
I. Water bodies				
Germany	Teltow Canal	Surface water	0.01–95.8 items/L	[49]
Denmark	Stormwater pond	Pond water	2.7 × 10^5^ items/m^3^	[50]
Portugal	Antuã River	Surface water	March 58–193 and October 71–1265 items/m^3^	[51]
South Korea	Korean coastal water	Surface water	1051 particles/m^3^	[52]
	Jinhae Bay	“	88 particles/L	[53]
USA	Lakes Huron	“	6541 particles/km^2^	[54]
	Hudson River	“	0.625–2.45 fibers/L	[55]
	Lake Superior	“	12,645 particles/km^2^	[54]
Europe	Carpathian Basin	“	3.52–32.05 particles/m^3^	[56]
	Rhine River	“	3.9 million particles/km^2^	[57]
England	Tamar Estuary	“	0.028 particles/m^3^	[58]
Qatar	Arabian Bay	“	4.38 × 10^4^–1.46 × 10^6^ particles/km^2^	[59]
Iran	Bandar Abbas	Surface water	3252 particles/m^2^	[60]
Italy	Subalpine lakes	“	4000–57,000 particles/km^2^	[61]
South Africa	Southeastern coastline	“	257.9–1215 particles/m^3^	[62]
Mexico	Northern Gulf	“	4.8–18.4 particles/m^3^	[63]
China	Bei Lake	“	8925 items/m^3^	[29]
	Huanzi Lake	“	8550 items/m^3^	[29]
	Pearl River	“	94–2098 items/m^2^	[64]
	Xianjia Lake	“	3825 items/m^2^	[65]
II. Sediments				
USA	Estuaries	Sediment	Charleston 413.8 and Winyah Bay 221.0	[66]
Europe	Carpathian Basin	“	9.5 × 10^5^ items/kg dw	[50]
Italy	Tyrrhenian Sea	“	42–1069 items/kg dw	[67]
Germany	Main river	“	786–1368 particles/kg dw	[68]
	Rhine River	“	228–3763 particles/kg dw	[68]
United Kingdom	River Tame	“	165 particles/kg dw	[69]
Portugal	Antuã River	“	March 100–629 and October 18–514 items/kg dw	[51]
Denmark	Stormwater pond	“	9.5 × 10^5^ items/kg dw	[50]
South Korea	Nakdong River	“	1971 particles/kg dw	[70]
Spain	Canary Islands	“	2–115.5 items/m^2^	[71]
	Remote beach	“	36.3 g/m^2^	[72]
Iran	Persian Gulf	“	61 particles/kg dw	[73]
	Persian Gulf	“	2–1258 particles/kg dw	[74]
Russia	Beaches	“	1.3–36.3 items/kg dw	[75]
	Baltic Sea	“	34 items/kg dw	[76]
China	North Yellow Sea	“	499.76 items/kg dw	[77]
	Bohai Bay	“	96.7–333.3 and 56.7–113.3 items/kg dw	[78]
	Yellow Sea	“	60–240 items/kg dw	[30]
	Maowei Sea	“	520–940 items/kg dw	[79]
III. Wastewater treatment plants				
Region	Type	Concentration	Polymer type	References
USA	Secondary	1–30 particles/L	Fibers and particles	[80]
UK	“	0.25–8.7 particles/L	Flakes, fibers, film, beads, and foam	[81]
Germany	“	0.08–7.52 particles/L	PE and PP	[82]
Australia	“	0.48 particles/L	PET fibers and irregularly shaped PE particles	[83]
Canada	“	0.5 particles/L	Fibers and fragments	[84]
China	“	28.4 particles/L	Fibers and fragments	[85]
Finland	“	0.4–1 particles/L	PE particles	[86]

## 3. Detection and Identification of MNPs

Different types of plastics have different chemical and physical characteristics, which poses a challenge in establishing a universal accurate method for identification. Gravimetric analysis and visual inspection are used usually for the quantification and identification of MPs, even if chemical characterization is applied at later stages. Visual investigation, Fourier transform infrared spectroscopy (FTIR), and Raman spectroscopy are the conventional methods used for the detection of plastic particles [87]. Visual investigation allows the classification of plastic particles based on their physical characteristics, directly observed by microscope or using a fluorescent microscope. This method is considered, at present, to be the most applicable and widely available for plastic particle identification and quantification, and is usually used before further chemical characterization [87]. However, it is highly time-consuming and not accurate because of the wide variation in results produced by different observers. For instance, MPs visually detected in beach sediments by multiple observers showed wide variation within the detection range of 60–100%, due to differences in individual perception, fatigue, experience, and underestimation (e.g., avoiding all white fragments in a sample) or overestimation (e.g., by counting some biological materials) of certain MP particles [88]. For the identification of plastic particles and study of their chemical characteristics, Raman spectroscopy and FTIR are widely used [89,90]. Other new methods have been suggested that are characterized by cost-effectiveness and high efficiency, including FTIR combined with focal plane array (FPA) detection, Nile red (NR) staining, thermogravimetric analysis combined with differential scanning calorimetry (TGA-DSC), thermal extraction desorption–gas chromatography–mass spectrometry (TED-GC–MS), etc. Thus, the identification and detection of NPs are major challenges, even more than for MPs. Photothermal atomic force microscopy coupled with infrared spectroscopy (AFM-IR) or Raman spectroscopy can be used to analyze MNPs [91]. In addition, organisms that are sensitive to MNPs can be used as biosensors for MNP detection, which might have an impact on stress response genes and cell surface proteins [92,93]. Overall, exploring new analytical methods and instruments that can be coupled with existing instruments will overcome the recent issues associated with the characterization of different plastic particles.

## 4. Remediation Technologies for MNPs

**Figure 2:**
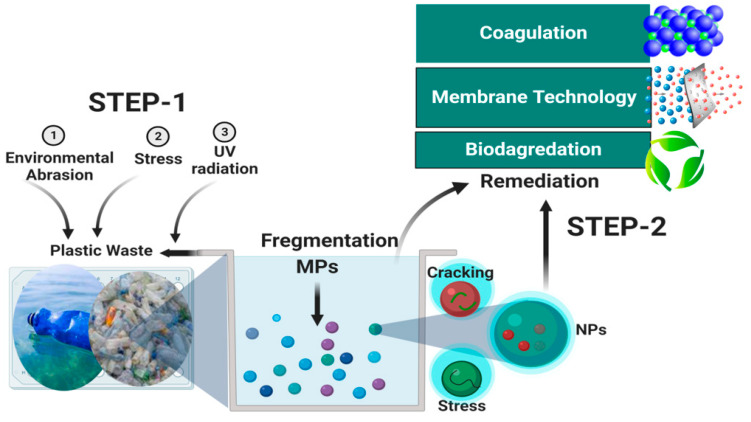
A schematic diagram showing the mechanism of plastic waste degradation to MNPs and the common remediation technologies.

Despite the current efforts in plastic management policies and the promotion of waste plastic recycling, improper plastic disposal is still the “*talk of the town*”. In the last decade, the removal of MNPs from aquatic environments represents a big challenge due to the concurrent disastrous impacts on aquatic species, humans, and the whole ecosystem [14]. So far, many remediation technologies and biotechnologies have been suggested for efficient waste plastic management [94,95,96], such as coagulation, membrane separation, and biodegradation (Figure 2).

### 4.1. Coagulation

The untreated sludge from WWTPs, which is contaminated with MNPs, is used as a biofertilizer in several countries, acting as a prime vehicle for the redistribution of MNPs into terrestrial and agroecosystems [97]. In WWTPs, MNP removal is usually carried out during the coagulation process [98], in which Al and Fe salts bind with plastic particles and facilitate their remediation through complexation [99]. Several studies have confirmed the efficiency of MNP removal via coagulation [95,100,101]. For instance, Ma et al. [95,100] evaluated coagulation efficiency using aluminum chloride and ferric chloride for the removal of PE particles of different sizes. The results confirmed the efficiency of plastic removal by coagulation in the case of relatively small particles, while particles of ≥5 mm were bigger than the typical colloidal particles removed by coagulation. Previous studies have evaluated MP removal by coagulation and subsequent ultrafiltration [95,100]. The results showed that the traditional coagulation process has low PE removal efficiency (below 15%) and that it is significantly affected by water characteristics. Thus, MP removal by ultrafiltration processes was suggested as a promising alternative for further application in drinking water treatment.

### 4.2. Membrane Technology

Although the removal of MNPs using membrane technology is still limited, recent years have seen a tremendous increase in the number of studies related to membrane bioreactors (MBRs) and the conventional membrane separation process for effective wastewater treatment coupled with energy production [14,102,103,104]. In addition, ultrafiltration using membrane separation has been recommended as an effective method for the removal of high MP concentrations which allows the attainment of high-quality drinking water with a relatively low energy consumption, high separation efficiency, and compact plant size [14]. For example, the utilization of an MBR improved the removal efficiency of MPs by up to 99.4% compared to conventional activated-sludge-based treatment [105]. The application of MBRs for MP and NP remediation in WWTPs revealed their superiority to oxidation ditches [106], which can be attributed to their dual action for anti-fouling and separation performance [107]. Although ultrafiltration coupled with coagulation is currently used in WWTPs and allows for significant removal of organic matter, this technology is not properly designed yet for the removal of MNPs that remain in the final effluents [108,109]. Thus, low concentrations of MPs can be detected in drinking water after treatment by MBR, which is attributed mainly to irreversible membrane fouling [100]. It was reported that ultrafiltration can be used to totally remove PE particles [14], but more research efforts and studies are still needed to understand how fouling and cake formation in MBRs are influenced by different loads of MNPs. In addition, the impacts of plastic shape on the removal process and reactor performance need to be evaluated. Moreover, the rate of MNP removal depends on hydrophobic and electrostatic interactions [110]; therefore, membrane-based technology requires further R&D for the adoption of measures and methods to overcome MNP-induced fouling to effectively remediate MNPs.

### 4.3. Biodegradation

Biodegradation is a new strategy for plastic waste remediation that has been increasingly discussed as an eco-friendly technology. Microbial potential (mainly bacteria and fungi) for plastic degradation through enzymatic hydrolysis has been intensively studied in recent years [111,112,113]. The exploration of new microbial enzymes and further mechanistic elucidations are crucial for enhanced MNP remediation by biodegradation [114]. Thus, identifying plastic-active enzymes for further application in biotechnological processes and elucidating their actual action in nature is an emerging research field, which is still in its infancy [36]. The process of MNP biodegradation is divided into four main steps [115]: biofilm development on plastispheres that decreases the hydrophobicity and buoyancy of plastic particles, followed by biodeterioration through exopolysaccharides and enzymatic action (endo-/exoenzymes). The third step is the destabilization of the carbon skeleton in MNP particles through enzymatic depolymerization, using oxidases, amidases, peroxidases, and laccases, then assimilation of monomers by microbial biomass [116]. Hydrolases were reported to play a vital role in plastic polymer hydrolysis and therefore in determining MNP biodegradation rates [117].

Certain microbes, such as the alkane-degrading marine bacterium *Alcanivorax borkumensis*, were reported to have a key role in LDPE degradation [118], resulting in significant physicochemical alterations. In addition, other bacterial strains, such as *Bacillus gottheilii* and *Bacillus cereus*, showed high potential to effectively remove a wide range of MNPs (PP, PET, PS, and PE) from mangrove sediments [119]. Fungi, such as *Zalerion maritimum*, also showed high efficiency in plastic degradation [120] using different mechanisms, through the release of sticky natural biosurfactants, such as hydrophobins [121]. In some cases, pretreatment is required to enhance the degradation process. In this context, PP biodegradation by two different fungal strains (*Phanerochaete chrysosporium* and *Engyodontium album*) was enhanced after starch/pro-oxidant pretreatment [122]. Despite their promising roles, the enzymes available at present act mainly on high-molecular-weight polymers of PET and ester-based PUR, with moderate turnover rates, and no enzymes acting on other high-molecular-weight polymers, such as PS, PP, ether-based PUR, and polyethylene are known [123]. Bioengineering of bacterial strains for enhanced protein production could further enhance MNP biodegradation by increasing enzyme activities [124]. In addition, the application of targeted microbial strain engineering can accelerate cellular enzymatic activities towards enhanced plastic degradation. In this respect, an engineered strain of *Bacillus subtilis* showed enhanced PETase activity (ca. four-fold) by inactivating the twin arginine translocation complexes, which further enhanced MNP degradation [125]. An integrated microalgae–bacteria system also showed potential to enhance the degradation of MNPs through enzymatic action. In this context, the photosynthetic diatom *Phaeodactylum tricornutum* was used as a cell factory for engineered PETase isolated from *Ideonella sakaiensis,* a known bacterium with a high capability for plastic degradation and for consuming it as both a carbon and energy source [126]. Although microbial biodegradation of MNPs is a promising approach, few studies have been conducted on microalgae, which require further investigations.

## 5. Plastic Waste and Microalgae as Biofuel Feedstocks

Due to modernization and industrialization, energy demand is increasing all over the world, giving rise to the need to overexploit the limited available natural resources for energy generation [127,128]. Currently, it is estimated that 524 quadrillion thermal British units (Btu) are consumed globally, which is projected to increase to 820 quadrillions Btu by 2040 [129]. Bioenergy can be produced from various biomass resources in the form of biodiesel, biogas, bioethanol, biohydrogen, and crude bio-oil. In this context, using first-generation biofuel feedstocks, which include edible food sources, such as soybean, rapeseed, sunflower, and corn, competes with human requirements for food and agricultural land, which raises the *Food-versus-Fuel* dispute [130]. To overcome such issues, the most effective techniques were developed to utilize waste and non-edible biomass. In this context, integrated approaches for the utilization of waste plastic [131,132,133] and/or microalgal biomass [134,135] as promising feedstocks for biofuel production have been discussed.

Waste plastic conversion involves the treatment of plastic waste to transform it into different forms of energy, including heat, electricity, and liquid fuels [136]. Plastic can be converted into different forms of biofuel via thermochemical conversion methods, including gasification, pyrolysis, and liquefaction. Algal biomass, meanwhile, can be converted into different forms of biofuel, including crude bio-oil, bioethanol, biogas, biodiesel, and bio-hydrogen [137,138,139], as well as value-added products/chemicals [140]. Compared to terrestrial plants and seaweeds, microalgal cells can accumulate more lipids over a shorter life cycle [139], and therefore they are discussed as a promising feedstock for third-generation biodiesel. In addition, microalgal biomass has been recognized as a carbon-neutral feedstock for fuel production due to its diverse phytochemical biomass characteristics, with high CO_2_ fixation efficiency. Thus, the development of microalgal biorefinery systems and the establishment of integrated routes have the potential to successfully reduce the reliance on fossil fuels and achieve a reduction in greenhouse gas (GHG) emissions, which would serve to mitigate the associated concerns about global warming and climate change.

## 6. Microalgae–MNP Interaction

Different from bacteria and fungi, microalgae are photoautotrophic organisms that can grow also mixotrophically in varied habitats, including water (fresh, marine, as well as wastewater), soil, and wet surfaces [141]. Microalgae have much higher biomass productivity compared to terrestrial plants [142], with a high capacity for the removal of heavy metals, ions, pesticides, pharmaceuticals, and other harmful contaminants. Different methods are used by microalgae to remove contaminants, such as adsorption, accumulation, and immobilization, followed by intracellular conversion to valuable products [143,144]. In WWTPs, there is a useful symbiotic interaction between microalgae and bacteria for pollutant removal. In these systems, autotrophic microalgae and heterotrophic bacteria rely on each other to grow, i.e., algal cells produce oxygen while bacterial cells use it for BOD removal and produce CO_2_ which is fixed by algal cells. In addition, the produced inorganic nitrogen and phosphorus are used by the microalgae for biomass production [145]. In lab experiments, microalgae showed high potential to treat different wastewater streams, such as municipal wastewater [146,147,148], distillery wastewater [149], brewery wastewater [150], pharmaceutical-rich wastewater [151], and dairy effluents [152]. In addition, microalgae have the potential to play a significant role in seawater desalination coupled with biofuel production [153,154,155]. From an economic perspective, microalgae have been reported as potential candidates to contribute to the bioeconomy through biofuel generation coupled with eco-friendly clean-up of different wastewaters and the application of biomass/byproducts as biofertilizers, nutrients, biopesticides, and bioplastics [156]. Compared to traditional biological wastewater treatment systems, microalgae offer many attractive benefits, such as cost-effectiveness, low energy consumption, higher pollutant removal, valuable biomass formation, nutrient recycling, and reductions in greenhouse gas emissions [157,158]. Thus, a microalgae-based system for MNP removal could have superior advantages over other biodegradation processes.

**Figure 3:**
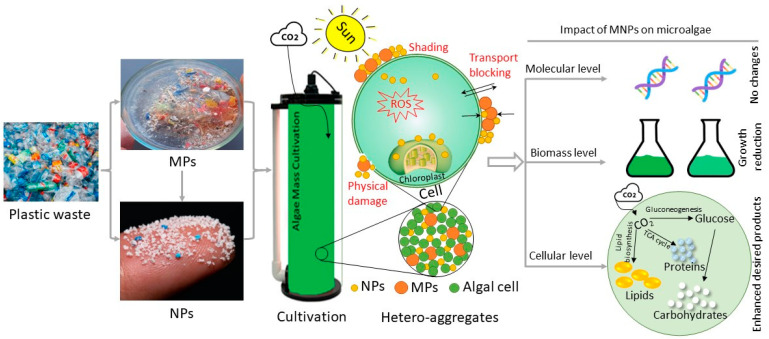
The modes of action and effects of microplastics (MPs) and nanoplastics (NPs) on microalgal cells and biomass production.

On the other hand, microalgae exist within a broad range of marine organisms that can be affected by MNPs, which alarms the scientific community due to the extreme importance of marine organisms as primary producers in the food chain [159,160]. Microalgae have a great capacity to interact with plastic particles in the aquatic system. Lagarde et al. [161] evaluated the interactions of PP and HDPE microplastics with the chlorophyte *Chlamydomonas reinhardtii* as a model microalgal species and observed a significant reduction in microalgal growth (about 18%) after 78 days of contact with PP at a concentration of 400 mg/L. This was attributed to the formation of hetero-aggregates of microalgae with microplastics over 20 days of mixing, which continued to increase until the end of the experiment. Microalgae trapping in MP aggregates explains the growth reduction in microalgae due to the reduction of photosynthetic efficiency because of shading effects [162]. However, the results showed no significant changes in the expression of the studied chloroplast genes using PP or HDPE and compared to the control (Figure 3) and thereby highlighted the negligible effect of plastics on microalgal molecular structure. Interestingly, HDPE at the applied high concentration of 400 mg/L showed a real effect on microalgal growth in a long-term experiment, while stress conditions applied in the experiment due to MNPs could enhance the production of desired compounds, such as lipids and carbohydrates (Figure 3), which could be beneficial for further biodiesel or bioethanol production.

Interactions between microalgae and MNPs may vary based on the cellular characteristics of the microalgae, such as shape, size, and physiological activity. In addition, the algal cell wall acts as a barrier to prevent particle penetration into the cell, and therefore cell wall characteristics influence MP sorption. However, NPs can easily penetrate the cell wall and might have an impact on algal cellular behavior. A previous study evaluated the response of the marine diatom *Thalassiosira pseudonana* with a silicate cell wall, the marine chlorophyte *Dunaliella tertiolecta* without a cell wall, and the freshwater chlorophyte *Chorella vulgaris* with a polysaccharidic cell wall to polystyrene particles [163]. Both negatively charged and uncharged particles of three different sizes (0.05, 0.5, and 6 μm) were tested. The results showed negligible effects on the photosynthetic efficiency of *D. tertiolecta* (<10% inhibition compared to the control) upon exposure to any of the three sizes of polystyrene beads, and none of the beads affected microalgal photosynthesis, even at the highest concentration of 250 mg polystyrene/L. However, microalgal growth was negatively affected (by up to 45%) by uncharged polystyrene, but only at high concentrations of 250 mg/L. The recorded negative impacts on growth were demonstrated to increase with decreasing particle size, which could be attributed to the possibility of cell wall/membrane penetration.

Different suggested mechanisms for the effects of MNPs on molecular and cellular levels are presented in Figure 3. MNPs could reduce photosynthetic activity and/or transportation mechanisms through accumulation at the cell surface. Additionally, MNPs can result in physical damage to the cell wall [164]. NPs can penetrate the cell and result in direct effects on chloroplasts, as well as other cellular organelles, and enhance reactive oxygen species (ROS) generation. As shown in Table 2, there is a confirmed influence of MNPs on microalgal growth, while no consistent conclusion can be summarized, because different studies were performed with different microalgal species, types of MNPs, and concentrations. For instance, recent studies have reported the negative impact of NPs on photosynthetic activity [165,166,167], while others have shown insignificant effects [163]. In addition, the type of MNP surface charge affects the inhibition level. Since microalgal cells have negative surface charges [168], positively charged MNPs have stronger interactions with microalgal cells due to electrostatic interactions. In this context, the growth of *Mycrocystis aeruginosa* was inhibited by 23.57% and 46.10%, respectively, after exposure to positively charged PS particles at concentrations of 3.40 and 6.8 mg/L [169]. However, exposure to negatively charged PS particles, even at a high concentration of 100 mg/L, showed insignificant effects on growth.

Due to the adsorption of other contaminants in the environment by MNP particles, there are confirmed synergistic and/or antagonistic interactions between MNPs and other contaminants. For instance, the combined action of NPs with dibutyl phthalate on *Chlorella pyrenoidosa* was evaluated [170]. The results showed that low NP concentrations of less than 10 mg/L resulted in antagonistic action at low dibutyl phthalate concentrations, while synergistic action was recorded at relatively high dibutyl phthalate concentrations. However, high NP concentrations of more than 10 mg/L resulted in antagonistic action with NPs. This was attributed to the competitive adsorption of dibutyl phthalate by NPs, which leads to reduction in dibutyl phthalate bioavailability. In conclusion, the impact of MNPs on the growth and photosynthetic activity of microalgae is species-dependent and also depends on the kind and size of particles used. Most studies that have evaluated MNP interactions with microalgae were short-term, while the long-term effects of MNPs on microalgal cells require further evaluation in order to elucidate the possibility of chronic effects or the adaptability of microalgae to MNPs. In addition, further studies should be conducted to explore the relationships between MNP characteristics and macromolecular changes in microalgal cells.

**Table 2:** Previous reports on the impact of micro-/nanoplastics (MNPs) on the growth and photosynthetic activity of microalgae.

Microalgae	MNPs			Impacts		Ref.
	Types	Size (nm)	Concentration (mg/L)	On Growth	On Photosynthesis	
*Skeletonema costatum*	PS, PE, and PVC	74,000	10, 20, 50, and 100	Growth inhibition	-	[171]
*Chlorella* *pyrenoidosa*	PS	100	10, 50, and 100	Dose-dependent negative effect from the lag to earlier logarithmic phase		[165]
*Scenedesmus obliquus*	PS	100 and 500	0 to 100	Significant inhibition	Significant inhibition	[172]
*Raphidocelis subcapitata*	PE	63,000–75,000	25, 50, and 100	Growth promotion	-	[173]
*Chaetoceros neogracile*	PS-NH_2_	500	2.5	No toxicity effect		[174]
*Dunaliella tertiolecta*	PS	50 and 500	25 and 250	Negative effect, adverse effects increase with decreasing particle size	No effect on microalgal photosynthesis	[163]
*Chlorella pyrenoidosa*	PS	100 and 550	0.5–64	Size-dependent inhibition effect; smaller size led to higher inhibition	Inhibition effect on chlorophyll fluorescence intensity	[170]
*Karenia mikimotoi*	PVC	1000	5, 25, 50, and 100	Growth inhibition	-	[175]
*Dunaliella salina*	PE	200,000	200, 250, 300, and 350	Growth promotion	-	[176]
*Phaeodactylum tricornutum*	PP, PE, PET, and PVC	74,000	200	Growth inhibition	-	[177]
*Chlorella* sp.	PE, PET, and PVC	74,000	200	Growth promotion	-	[177]

A recent study confirmed that MPs induce nutrient and environmental stress, which further enhances lipid accumulation and the production of other desirable macromolecules [178]. In this regard, several pathways have been suggested for biofuel production using microalgae based on the characteristics of the produced biomass, including bioethanol production through fermentation, direct transesterification of lipids into biodiesel, anaerobic digestion for biogas production, and thermochemical conversion for crude bio-oil production [179]. Microalgal lipids and carbohydrates have been discussed as potential feedstocks for biodiesel and bioethanol/biobutanol, respectively [180,181,182,183,184]; however, such individual routes showed relatively low energy recovery due to partial conversion of the biomass to energy, which results in an elevated cost for the final product [185]. Therefore, the large-scale production of biofuel from microalgae has not yet been realized. Major R&D gaps, such as maximizing energy yield, reducing energy input, and cost-effective cultivation, need to be addressed. Regarding energy, sequential biofuel recovery using two or more conversion methods has recently been suggested in order to enhance energy recovery from microalgal biomass. For instance, the sequential biodiesel and biogas route enables the initial utilization of lipids for biodiesel production, followed by anaerobic digestion of lipid-free residual biomass for biogas production, which increases the overall energy output [186]. In addition, there is an integrated route of sequential fermentation of *Chlamydomonas mexicana* biomass to carbohydrates and proteins, followed by lipid transesterification, then further fermentation of lipid-free residue and waste glycerol [187]. The utilization of such a high-throughput sequential route resulted in plenty of biofuels, including higher alcohols (from proteins), bioethanol (from carbohydrates), biobutanol (from lipid-free residues with glycerol), along with biodiesel, leading to a high biomass conversion efficiency of 89%. Regarding cost-effective cultivation, the cost of growth medium for nutrient supplementation has a significant impact on the overall production cost, whereas major nutrients can be delivered from wastewater. In addition, some mixotrophic microalgae might be able to ingest/degrade MNPs in wastewater, which requires further validation through screening studies. Such integrated approaches could have significant impacts in terms of the maximization of dual microalgae utilization for MNP removal coupled with energy production.

## 7. MNP–Microalgae Biofuel Integrated Approach

**Figure 4:**
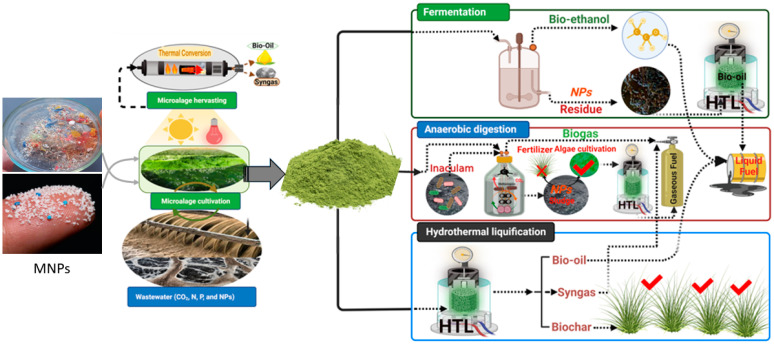
The suggested microalgae–MNP integrated routes for biofuel production using wastewater for cultivation. HTL: hydrothermal liquefaction; MNPs: micro-/nanoplastics.

Microalgal biomass generation using wastewater as a growth medium and/or flue gas for CO_2_ supplementation has been considered advantageous in terms of waste and emission reduction while producing valuable biomass, leading to sustainable development [185]. Another aspect of biofuel production from microalgal biomass is maximum energy recovery through whole biomass conversion to achieve a zero-waste approach, as discussed in the previous section. The aforementioned two aspects of microalgae cultivation could further enhance their potential, which supports sustainable fuel production coupled with other applications and reduced emissions. However, the main bottlenecks for establishing a microalgal-based biofuel system are the elevated production costs and the fact that integrated microalgal biorefinery routes are in their infancy [185]. On the other hand, serious measures have been implemented to manage plastics, from production to disposal. For waste plastic disposal, four main methods are currently used globally, namely, landfill, incineration, thermochemical conversion, and regenerative granulation [188]. Among them, thermochemical conversion into biofuel is recommended to avoid landfill constraints, reduce costs, and provide carbon neutrality [189]. Compared with other thermochemical conversion methods, hydrothermal liquefaction was suggested for microalgal biomass conversion in order to avoid the drying step and reduce the energy input. Plastic waste can also be blended with biomass and undergo hydrothermal co-liquefaction, which produces syngas, biochar, and bio-oil of better quality than the conversion of individual feedstocks due to synergistic action [190]. Other biological conversion methods, such as fermentation and anaerobic digestion of microalgal biomass, can also be utilized [191,192]. Regarding the biomass harvested after MNP absorption from contaminated water, special precautions are needed to avoid the re-entry of MNPs into the ecosystem. Hence, a consolidated approach of sequential biomass conversion, including thermal conversion for MNP degradation, has been suggested to achieve finite improvements in terms of energy and the environment. From an economic perspective, biomass biorefineries, through maximizing energy recovery by full utilization of biomass, could be cost-effective systems, and there is much encouragement nowadays of research and development. In this regard, microalgal biomass contains a magnificent amount of energy, which can be significantly utilized through different conversion pathways (Figure 4). Previous studies have shown that the conversion of microalgae to crude bio-oil has the highest energy output among different pathways, due to the advantage that bio-oil production involves the conversion of whole biomass rather than the conversion of a particular component [185]. In addition, the thermal conversion of MNP-contaminated microalgal biomass could serve the aim of the safe disposal of MNP particles. As previously discussed, MNP particles can also undergo hetero-aggregation with microalgae, which would be a promising approach for possible application in microalgae harvesting. In addition, low concentrations of MNPs could enhance the accumulation of desirable macromolecules, such as lipids and/or carbohydrates, based on the microalgal species. Thus, microalgae–MNP-based biorefining through integrated conversion routes, could present a new approach to be investigated, with the aim of providing a wide range of products, including treated water, biofuels, biopolymers, and biofertilizers, all of which are in high demand globally. In summary, microalgae can grow in MNP-contaminated water and act as bio-scavengers for plastic particles. Pyrolysis and/or hydrothermal liquefaction can be applied to MNP-rich microalgal biomass for crude biofuel production in the form of syngas and crude bio-oil (Figure 4). The biochar so produced can be used for many purposes, mainly as a soil amendment or in the development of biocatalysts, which can be further employed for enhanced biofuel production. Further studies are required to evaluate the impacts of MNPs on biological conversion processes, such as anaerobic digestion and fermentation of MNP-contaminated biomass, for biogas and bioethanol production. The digested residue after biological conversion rich in MNPs can undergo hydrothermal liquefaction, ensuring a zero-waste approach.

## 8. Conclusions

There is a vital need to explore sustainable and alternative energy forms, which is steadily growing with the increasing depletion of fossil fuel resources. In addition, MNP distribution in all water bodies is alarming, and serious measures need to be taken to avoid the negative impacts. The utilization of microalgae as biofuel feedstocks offers an economic and eco-friendly alternative to the use of fossil fuels. It also could serve the aim of wastewater treatment and MNP removal. Interactions between MNPs and microalgal cells could enhance several important features for possible microalgal harvest and/or desirable macromolecule accumulation. One hypothesis is that microalgal biomass can accumulate lipids and carbohydrates under MNP stress, supporting biomass conversion into biodiesel and bioethanol, respectively. In addition, microalgal biomass can be converted to biogas through anaerobic digestion. However, biological conversion results in residues rich in MNPs, which are associated with serious concerns about plastic redistribution into the environment. The most recommended route in such cases is thermochemical conversion, which could be considered as a post-treatment process for microplastic conversion as well. In such systems, algal cells act as bio-scavengers for MNPs, binding the particles to algal surfaces or incorporating them into their cells so that they are filtered from the water body and finally destroyed by further downstream processing of the polluted biomass. The present article has suggested a new approach that could assist in MNP removal from contaminated water through microalgal cultivation together with sustainable biofuel production using a net-zero approach in order to mitigate the environmental impacts of disposed waste.

## References

[1] Suzuki G., Uchida N., Tuyen L.H., Tanaka K., Matsukami H., Kunisue T., Takahashi S., Viet P.H., Kuramochi H., Osako M. (2022). Mechanical Recycling of Plastic Waste as a Point Source of Microplastic Pollution. Environ. Pollut..

[2] The World Bank Global Waste to Grow by 70 Percent by 2050 Unless Urgent Action Is Taken: World Bank Report. https://www.worldbank.org/en/news/press-release/2018/09/20/global-waste-to-grow-by-70-percent-by-2050-unless-urgent-action-is-taken-world-bank-report.

[3] Geyer R., Jambeck J.R., Law K.L. (2017). Production, Use, and Fate of All Plastics Ever Made. Sci. Adv..

[4] PlasticsEurope (2018). Plastics–The Facts 2018: An Analysis of European Plastics Production, Demand and Waste Data.

[5] Astner A.F., Hayes D.G., O’Neill H., Evans B.R., Pingali S.V., Urban V.S., Young T.M. (2019). Mechanical Formation of Micro- and Nano-Plastic Materials for Environmental Studies in Agricultural Ecosystems. Sci. Total Environ..

[6] Ng E.L., Huerta Lwanga E., Eldridge S.M., Johnston P., Hu H.W., Geissen V., Chen D. (2018). An Overview of Microplastic and Nanoplastic Pollution in Agroecosystems. Sci. Total Environ..

[7] Song Z., Yang X., Chen F., Zhao F., Zhao Y., Ruan L., Wang Y., Yang Y. (2019). Fate and Transport of Nanoplastics in Complex Natural Aquifer Media: Effect of Particle Size and Surface Functionalization. Sci. Total Environ..

[8] Wang L., Wu W.M., Bolan N.S., Tsang D.C.W., Li Y., Qin M., Hou D. (2021). Environmental Fate, Toxicity and Risk Management Strategies of Nanoplastics in the Environment: Current Status and Future Perspectives. J. Hazard. Mater..

[9] Yin K., Wang Y., Zhao H., Wang D., Guo M., Mu M., Liu Y., Nie X., Li B., Li J. (2021). A Comparative Review of Microplastics and Nanoplastics: Toxicity Hazards on Digestive, Reproductive and Nervous System. Sci. Total Environ..

[10] Schwaferts C., Niessner R., Elsner M., Ivleva N.P. (2019). Methods for the Analysis of Submicrometer- and Nanoplastic Particles in the Environment. TrAC Trends Anal. Chem..

[11] Oliveira M., Almeida M., Miguel I. (2019). A Micro(Nano)Plastic Boomerang Tale: A Never Ending Story?. TrAC Trends Anal. Chem..

[12] Ferreira I., Venâncio C., Lopes I., Oliveira M. (2019). Nanoplastics and Marine Organisms: What Has Been Studied?. Environ. Toxicol. Pharmacol..

[13] Koelmans A.A., Besseling E., Shim W.J. (2015). Nanoplastics in the Aquatic Environment. Critical Review. Mar. Anthropog. Litter.

[14] Poerio T., Piacentini E., Mazzei R. (2019). Membrane Processes for Microplastic Removal. Molecules.

[15] Barría C., Brandts I., Tort L., Oliveira M., Teles M. (2020). Effect of Nanoplastics on Fish Health and Performance: A Review. Mar. Pollut. Bull..

[16] Antranikian G., Streit W.R. (2022). Microorganisms Harbor Keys to a Circular Bioeconomy Making Them Useful Tools in Fighting Plastic Pollution and Rising CO_2_ Levels. Extremophiles.

[17] Cole M., Lindeque P., Fileman E., Halsband C., Goodhead R., Moger J., Galloway T.S. (2013). Microplastic Ingestion by Zooplankton. Environ. Sci. Technol..

[18] Nizzetto L., Futter M., Langaas S. (2016). Are Agricultural Soils Dumps for Microplastics of Urban Origin?. Environ. Sci. Technol..

[19] Eriksen M., Lebreton L.C.M., Carson H.S., Thiel M., Moore C.J., Borerro J.C., Galgani F., Ryan P.G., Reisser J. (2014). Plastic Pollution in the World’s Oceans: More than 5 Trillion Plastic Pieces Weighing over 250,000 Tons Afloat at Sea. PLoS ONE.

[20] Akdogan Z., Guven B. (2019). Microplastics in the Environment: A Critical Review of Current Understanding and Identification of Future Research Needs. Environ. Pollut..

[21] Rochman C.M., Kross S.M., Armstrong J.B., Bogan M.T., Darling E.S., Green S.J., Smyth A.R., Veríssimo D. (2015). Scientific Evidence Supports a Ban on Microbeads. Environ. Sci. Technol..

[22] Hernandez L.M., Yousefi N., Tufenkji N. (2017). Are There Nanoplastics in Your Personal Care Products. Environ. Sci. Technol. Lett..

[23] Lechner A., Keckeis H., Lumesberger-Loisl F., Zens B., Krusch R., Tritthart M., Glas M., Schludermann E. (2014). The Danube so Colourful: A Potpourri of Plastic Litter Outnumbers Fish Larvae in Europe’s Second Largest River. Environ. Pollut..

[24] Alimi O.S., Farner Budarz J., Hernandez L.M., Tufenkji N. (2018). Microplastics and Nanoplastics in Aquatic Environments: Aggregation, Deposition, and Enhanced Contaminant Transport. Environ. Sci. Technol..

[25] Hidalgo-Ruz V., Gutow L., Thompson R.C., Thiel M. (2012). Microplastics in the Marine Environment: A Review of the Methods Used for Identification and Quantification. Environ. Sci. Technol..

[26] Xu S., Ma J., Ji R., Pan K., Miao A.J. (2020). Microplastics in Aquatic Environments: Occurrence, Accumulation, and Biological Effects. Sci. Total Environ..

[27] Wang J., Wang M., Ru S., Liu X. (2019). High Levels of Microplastic Pollution in the Sediments and Benthic Organisms of the South Yellow Sea, China. Sci. Total Environ..

[28] Pan Z., Guo H., Chen H., Wang S., Sun X., Zou Q., Zhang Y., Lin H., Cai S., Huang J. (2019). Microplastics in the Northwestern Pacific: Abundance, Distribution, and Characteristics. Sci. Total Environ..

[29] Wang W., Ndungu A.W., Li Z., Wang J. (2017). Microplastics Pollution in Inland Freshwaters of China: A Case Study in Urban Surface Waters of Wuhan, China. Sci. Total Environ..

[30] Zhang C., Zhou H., Cui Y., Wang C., Li Y., Zhang D. (2019). Microplastics in Offshore Sediment in the Yellow Sea and East China Sea, China. Environ. Pollut..

[31] Mintenig S.M., Löder M.G.J., Primpke S., Gerdts G. (2019). Low Numbers of Microplastics Detected in Drinking Water from Ground Water Sources. Sci. Total Environ..

[32] Cao Y., Zhao M., Ma X., Song Y., Zuo S., Li H., Deng W. (2021). A Critical Review on the Interactions of Microplastics with Heavy Metals: Mechanism and Their Combined Effect on Organisms and Humans. Sci. Total Environ..

[33] Güven O., Gökdağ K., Jovanović B., Kıdeyş A.E. (2017). Microplastic Litter Composition of the Turkish Territorial Waters of the Mediterranean Sea, and Its Occurrence in the Gastrointestinal Tract of Fish. Environ. Pollut..

[34] Lanctôt C.M., Al-Sid-Cheikh M., Catarino A.I., Cresswell T., Danis B., Karapanagioti H.K., Mincer T., Oberhänsli F., Swarzenski P., Tolosa I. (2018). Application of Nuclear Techniques to Environmental Plastics Research. J. Environ. Radioact..

[35] Wright R.J., Bosch R., Gibson M.I., Christie-Oleza J.A. (2020). Plasticizer Degradation by Marine Bacterial Isolates: A Proteogenomic and Metabolomic Characterization. Environ. Sci. Technol..

[36] Chow J., Perez-Garcia P., Dierkes R., Streit W.R. (2022). Microbial Enzymes Will Offer Limited Solutions to the Global Plastic Pollution Crisis. Microb. Biotechnol..

[37] Ašmonaite G., Larsson K., Undeland I., Sturve J., Carney Almroth B. (2018). Size Matters: Ingestion of Relatively Large Microplastics Contaminated with Environmental Pollutants Posed Little Risk for Fish Health and Fillet Quality. Environ. Sci. Technol..

[38] Rios Mendoza L.M., Karapanagioti H., Álvarez N.R. (2018). Micro (Nanoplastics) in the Marine Environment: Current Knowledge and Gaps. Curr. Opin. Environ. Sci. Health.

[39] Peng L., Fu D., Qi H., Lan C.Q., Yu H., Ge C. (2020). Micro- and Nano-Plastics in Marine Environment: Source, Distribution and Threats—A Review. Sci. Total Environ..

[40] Okoye C.O., Addey C.I., Oderinde O., Okoro J.O., Uwamungu J.Y., Ikechukwu C.K., Okeke E.S., Ejeromedoghene O., Odii E.C. (2022). Toxic Chemicals and Persistent Organic Pollutants Associated with Micro-and Nanoplastics Pollution. Chem. Eng. J. Adv..

[41] Chae Y., Kim D., Kim S.W., An Y.J. (2018). Trophic Transfer and Individual Impact of Nano-Sized Polystyrene in a Four-Species Freshwater Food Chain. Sci. Rep..

[42] Barboza L.G.A., Dick Vethaak A., Lavorante B.R.B.O., Lundebye A.K., Guilhermino L. (2018). Marine Microplastic Debris: An Emerging Issue for Food Security, Food Safety and Human Health. Mar. Pollut. Bull..

[43] Revel M., Châtel A., Mouneyrac C. (2018). Micro(Nano)Plastics: A Threat to Human Health?. Curr. Opin. Environ. Sci. Health.

[44] Schwabl P., Koppel S., Konigshofer P., Bucsics T., Trauner M., Reiberger T., Liebmann B. (2019). Detection of Various Microplastics in Human Stool: A Prospective Case Series. Ann. Intern. Med..

[45] Ibrahim Y.S., Tuan Anuar S., Azmi A.A., Wan Mohd Khalik W.M.A., Lehata S., Hamzah S.R., Ismail D., Ma Z.F., Dzulkarnaen A., Zakaria Z. (2021). Detection of Microplastics in Human Colectomy Specimens. JGH Open.

[46] Leslie H.A., van Velzen M.J.M., Brandsma S.H., Vethaak A.D., Garcia-Vallejo J.J., Lamoree M.H. (2022). Discovery and Quantification of Plastic Particle Pollution in Human Blood. Environ. Int..

[47] Ragusa A., Svelato A., Santacroce C., Catalano P., Notarstefano V., Carnevali O., Papa F., Rongioletti M.C.A., Baiocco F., Draghi S. (2021). Plasticenta: First Evidence of Microplastics in Human Placenta. Environ. Int..

[48] Braun T., Ehrlich L., Henrich W., Koeppel S., Lomako I., Schwabl P., Liebmann B. (2021). Detection of Microplastic in Human Placenta and Meconium in a Clinical Setting. Pharmaceutics.

[49] Schmidt L.K., Bochow M., Imhof H.K., Oswald S.E. (2018). Multi-Temporal Surveys for Microplastic Particles Enabled by a Novel and Fast Application of SWIR Imaging Spectroscopy–Study of an Urban Watercourse Traversing the City of Berlin, Germany. Environ. Pollut..

[50] Olesen K.B., Stephansen D.A., van Alst N., Vollertsen J. (2019). Microplastics in a Stormwater Pond. Water.

[51] Rodrigues M.O., Abrantes N., Gonçalves F.J.M., Nogueira H., Marques J.C., Gonçalves A.M.M. (2018). Spatial and Temporal Distribution of Microplastics in Water and Sediments of a Freshwater System (Antuã River, Portugal). Sci. Total Environ..

[52] Song Y.K., Hong S.H., Eo S., Jang M., Han G.M., Isobe A., Shim W.J. (2018). Horizontal and Vertical Distribution of Microplastics in Korean Coastal Waters. Environ. Sci. Technol..

[53] Song Y.K., Hong S.H., Jang M., Han G.M., Shim W.J. (2015). Occurrence and Distribution of Microplastics in the Sea Surface Microlayer in Jinhae Bay, South Korea. Arch. Environ. Contam. Toxicol..

[54] Eriksen M., Mason S., Wilson S., Box C., Zellers A., Edwards W., Farley H., Amato S. (2013). Microplastic Pollution in the Surface Waters of the Laurentian Great Lakes. Mar. Pollut. Bull..

[55] Miller R.Z., Watts A.J.R., Winslow B.O., Galloway T.S., Barrows A.P.W. (2017). Mountains to the Sea: River Study of Plastic and Non-Plastic Microfiber Pollution in the Northeast USA. Mar. Pollut. Bull..

[56] Bordós G., Urbányi B., Micsinai A., Kriszt B., Palotai Z., Szabó I., Hantosi Z., Szoboszlay S. (2019). Identification of Microplastics in Fish Ponds and Natural Freshwater Environments of the Carpathian Basin, Europe. Chemosphere.

[57] Mani T., Hauk A., Walter U., Burkhardt-Holm P. (2015). Microplastics Profile along the Rhine River. Sci. Rep..

[58] Sadri S.S., Thompson R.C. (2014). On the Quantity and Composition of Floating Plastic Debris Entering and Leaving the Tamar Estuary, Southwest England. Mar. Pollut. Bull..

[59] Abayomi O.A., Range P., Al-Ghouti M.A., Obbard J.P., Almeer S.H., Ben-Hamadou R. (2017). Microplastics in Coastal Environments of the Arabian Gulf. Mar. Pollut. Bull..

[60] Nabizadeh R., Sajadi M., Rastkari N., Yaghmaeian K. (2019). Microplastic Pollution on the Persian Gulf Shoreline: A Case Study of Bandar Abbas City, Hormozgan Province, Iran. Mar. Pollut. Bull..

[61] Sighicelli M., Pietrelli L., Lecce F., Iannilli V., Falconieri M., Coscia L., Di Vito S., Nuglio S., Zampetti G. (2018). Microplastic Pollution in the Surface Waters of Italian Subalpine Lakes. Environ. Pollut..

[62] Nel H.A., Froneman P.W. (2015). A Quantitative Analysis of Microplastic Pollution along the South-Eastern Coastline of South Africa. Mar. Pollut. Bull..

[63] Cincinelli A., Scopetani C., Chelazzi D., Lombardini E., Martellini T., Katsoyiannis A., Fossi M.C., Corsolini S. (2017). Microplastic in the Surface Waters of the Ross Sea (Antarctica): Occurrence, Distribution and Characterization by FTIR. Chemosphere.

[64] Fok L., Cheung P.K. (2015). Hong Kong at the Pearl River Estuary: A Hotspot of Microplastic Pollution. Mar. Pollut. Bull..

[65] Yin L., Jiang C., Wen X., Du C., Zhong W., Feng Z., Long Y., Ma Y. (2019). Microplastic Pollution in Surface Water of Urban Lakes in Changsha, China. Int. J. Environ. Res. Public Health.

[66] Gray A.D., Wertz H., Leads R.R., Weinstein J.E. (2018). Microplastic in Two South Carolina Estuaries: Occurrence, Distribution, and Composition. Mar. Pollut. Bull..

[67] Cannas S., Fastelli P., Guerranti C., Renzi M. (2017). Plastic Litter in Sediments from the Coasts of South Tuscany (Tyrrhenian Sea). Mar. Pollut. Bull..

[68] Klein S., Worch E., Knepper T.P. (2015). Occurrence and Spatial Distribution of Microplastics in River Shore Sediments of the Rhine-Main Area in Germany. Environ. Sci. Technol..

[69] Tibbetts J., Krause S., Lynch I., Smith G.H.S. (2018). Abundance, Distribution, and Drivers of Microplastic Contamination in Urban River Environments. Water.

[70] Eo S., Hong S.H., Song Y.K., Han G.M., Shim W.J. (2019). Spatiotemporal Distribution and Annual Load of Microplastics in the Nakdong River, South Korea. Water Res..

[71] Álvarez-Hernández C., Cairós C., López-Darias J., Mazzetti E., Hernández-Sánchez C., González-Sálamo J., Hernández-Borges J. (2019). Microplastic Debris in Beaches of Tenerife (Canary Islands, Spain). Mar. Pollut. Bull..

[72] Edo C., Tamayo-Belda M., Martínez-Campos S., Martín-Betancor K., González-Pleiter M., Pulido-Reyes G., García-Ruiz C., Zapata F., Leganés F., Fernández-Piñas F. (2019). Occurrence and Identification of Microplastics along a Beach in the Biosphere Reserve of Lanzarote. Mar. Pollut. Bull..

[73] Naji A., Esmaili Z., Mason S.A., Dick Vethaak A. (2017). The Occurrence of Microplastic Contamination in Littoral Sediments of the Persian Gulf, Iran. Environ. Sci. Pollut. Res..

[74] Naji A., Esmaili Z., Khan F.R. (2017). Plastic Debris and Microplastics along the Beaches of the Strait of Hormuz, Persian Gulf. Mar. Pollut. Bull..

[75] Esiukova E. (2017). Plastic Pollution on the Baltic Beaches of Kaliningrad Region, Russia. Mar. Pollut. Bull..

[76] Zobkov M., Esiukova E. (2017). Microplastics in Baltic Bottom Sediments: Quantification Procedures and First Results. Mar. Pollut. Bull..

[77] Zhang B., Wu D., Yang X., Teng J., Liu Y., Zhang C., Zhao J., Yin X., You L., Liu Y. (2019). Microplastic Pollution in the Surface Sediments Collected from Sishili Bay, North Yellow Sea, China. Mar. Pollut. Bull..

[78] Wu N., Zhang Y., Zhang X., Zhao Z., He J., Li W., Ma Y., Niu Z. (2019). Occurrence and Distribution of Microplastics in the Surface Water and Sediment of Two Typical Estuaries in Bohai Bay, China. Environ. Sci. Process. Impacts.

[79] Li R., Zhang L., Xue B., Wang Y. (2019). Abundance and Characteristics of Microplastics in the Mangrove Sediment of the Semi-Enclosed Maowei Sea of the South China Sea: New Implications for Location, Rhizosphere, and Sediment Compositions. Environ. Pollut..

[80] Conley K., Clum A., Deepe J., Lane H., Beckingham B. (2019). Wastewater Treatment Plants as a Source of Microplastics to an Urban Estuary: Removal Efficiencies and Loading per Capita over One Year. Water Res. X.

[81] Murphy F., Ewins C., Carbonnier F., Quinn B. (2016). Wastewater Treatment Works (WwTW) as a Source of Microplastics in the Aquatic Environment. Environ. Sci. Technol..

[82] Mintenig S.M., Int-Veen I., Löder M.G.J., Primpke S., Gerdts G. (2017). Identification of Microplastic in Effluents of Waste Water Treatment Plants Using Focal Plane Array-Based Micro-Fourier-Transform Infrared Imaging. Water Res..

[83] Ziajahromi S., Neale P.A., Rintoul L., Leusch F.D.L. (2017). Wastewater Treatment Plants as a Pathway for Microplastics: Development of a New Approach to Sample Wastewater-Based Microplastics. Water Res..

[84] Gies E.A., LeNoble J.L., Noël M., Etemadifar A., Bishay F., Hall E.R., Ross P.S. (2018). Retention of Microplastics in a Major Secondary Wastewater Treatment Plant in Vancouver, Canada. Mar. Pollut. Bull..

[85] Liu X., Yuan W., Di M., Li Z., Wang J. (2019). Transfer and Fate of Microplastics during the Conventional Activated Sludge Process in One Wastewater Treatment Plant of China. Chem. Eng. J..

[86] Lares M., Ncibi M.C., Sillanpää M., Sillanpää M. (2018). Occurrence, Identification and Removal of Microplastic Particles and Fibers in Conventional Activated Sludge Process and Advanced MBR Technology. Water Res..

[87] Prata J.C., da Costa J.P., Duarte A.C., Rocha-Santos T. (2019). Methods for Sampling and Detection of Microplastics in Water and Sediment: A Critical Review. TrAC Trends Anal. Chem..

[88] Lavers J.L., Oppel S., Bond A.L. (2016). Factors Influencing the Detection of Beach Plastic Debris. Mar. Environ. Res..

[89] Elert A.M., Becker R., Duemichen E., Eisentraut P., Falkenhagen J., Sturm H., Braun U. (2017). Comparison of Different Methods for MP Detection: What Can We Learn from Them, and Why Asking the Right Question before Measurements Matters?. Environ. Pollut..

[90] Qiu Q., Tan Z., Wang J., Peng J., Li M., Zhan Z. (2016). Extraction, Enumeration and Identification Methods for Monitoring Microplastics in the Environment. Estuar. Coast. Shelf Sci..

[91] Eronen P., Österberg M., Jääskeläinen A.S. (2009). Effect of Alkaline Treatment on Cellulose Supramolecular Structure Studied with Combined Confocal Raman Spectroscopy and Atomic Force Microscopy. Cellulose.

[92] von der Kammer F., Ferguson P.L., Holden P.A., Masion A., Rogers K.R., Klaine S.J., Koelmans A.A., Horne N., Unrine J.M. (2011). Analysis of Engineered Nanomaterials in Complex Matrices (Environment and Biota): General Considerations and Conceptual Case Studies. Environ. Toxicol. Chem..

[93] Zhang Z., Chen Y. (2020). Effects of Microplastics on Wastewater and Sewage Sludge Treatment and Their Removal: A Review. Chem. Eng. J..

[94] Bornscheuer U.T. (2016). MICROBIOLOGY. Feeding on Plastic. Science.

[95] Ma B., Xue W., Hu C., Liu H., Qu J., Li L. (2019). Characteristics of Microplastic Removal via Coagulation and Ultrafiltration during Drinking Water Treatment. Chem. Eng. J..

[96] Wang L., Kaeppler A., Fischer D., Simmchen J. (2019). Photocatalytic TiO2 Micromotors for Removal of Microplastics and Suspended Matter. ACS Appl. Mater. Interfaces.

[97] Edo C., González-Pleiter M., Leganés F., Fernández-Piñas F., Rosal R. (2020). Fate of Microplastics in Wastewater Treatment Plants and Their Environmental Dispersion with Effluent and Sludge. Environ. Pollut..

[98] Skaf D.W., Punzi V.L., Rolle J.T., Kleinberg K.A. (2020). Removal of Micron-Sized Microplastic Particles from Simulated Drinking Water via Alum Coagulation. Chem. Eng. J..

[99] Chorghe D., Sari M.A., Chellam S. (2017). Boron Removal from Hydraulic Fracturing Wastewater by Aluminum and Iron Coagulation: Mechanisms and Limitations. Water Res..

[100] Ma B., Xue W., Ding Y., Hu C., Liu H., Qu J. (2019). Removal Characteristics of Microplastics by Fe-Based Coagulants during Drinking Water Treatment. J. Environ. Sci..

[101] Novotna K., Cermakova L., Pivokonska L., Cajthaml T., Pivokonsky M. (2019). Microplastics in Drinking Water Treatment–Current Knowledge and Research Needs. Sci. Total Environ..

[102] Pu Y., Tang J., Zeng T., Hu Y., Wang Q., Huang J., Pan S., Wang X.C., Li Y., Hao Ngo H. (2022). Enhanced Energy Production and Biological Treatment of Swine Wastewater Using Anaerobic Membrane Bioreactor: Fouling Mechanism and Microbial Community. Bioresour. Technol..

[103] Tang J., Pu Y., Zeng T., Hu Y., Huang J., Pan S., Wang X.C., Li Y., Abomohra A.E.F. (2022). Enhanced Methane Production Coupled with Livestock Wastewater Treatment Using Anaerobic Membrane Bioreactor: Performance and Membrane Filtration Properties. Bioresour. Technol..

[104] Pu Y., Tang J., Zeng T., Hu Y., Yang J., Wang X., Huang J., Abomohra A. (2022). Pollutant Removal and Energy Recovery from Swine Wastewater Using Anaerobic Membrane Bioreactor: A Comparative Study with Up-Flow Anaerobic Sludge Blanket. Water.

[105] Pico Y., Alfarhan A., Barcelo D. (2019). Nano- and Microplastic Analysis: Focus on Their Occurrence in Freshwater Ecosystems and Remediation Technologies. TrAC Trends Anal. Chem..

[106] Lv X., Dong Q., Zuo Z., Liu Y., Huang X., Wu W.M. (2019). Microplastics in a Municipal Wastewater Treatment Plant: Fate, Dynamic Distribution, Removal Efficiencies, and Control Strategies. J. Clean. Prod..

[107] Sun Z.K., Zhou Y., Jiao Y., Cheng X.Q., Zhang Y., Wang P., Liang H., Yang X., Drioli E., Figoli A. (2020). Multi-Hydrophilic Functional Network Enables Porous Membranes Excellent Anti-Fouling Performance for Highly Efficient Water Remediation. J. Membr. Sci..

[108] Talvitie J., Mikola A., Koistinen A., Setälä O. (2017). Solutions to Microplastic Pollution–Removal of Microplastics from Wastewater Effluent with Advanced Wastewater Treatment Technologies. Water Res..

[109] Mason S.A., Garneau D., Sutton R., Chu Y., Ehmann K., Barnes J., Fink P., Papazissimos D., Rogers D.L. (2016). Microplastic Pollution Is Widely Detected in US Municipal Wastewater Treatment Plant Effluent. Environ. Pollut..

[110] Enfrin M., Lee J., Le-Clech P., Dumée L.F. (2020). Kinetic and Mechanistic Aspects of Ultrafiltration Membrane Fouling by Nano- and Microplastics. J. Membr. Sci..

[111] Zhu L., Zhao S., Bittar T.B., Stubbins A., Li D. (2020). Photochemical Dissolution of Buoyant Microplastics to Dissolved Organic Carbon: Rates and Microbial Impacts. J. Hazard. Mater..

[112] Zhu K., Jia H., Sun Y., Dai Y., Zhang C., Guo X., Wang T., Zhu L. (2020). Long-Term Phototransformation of Microplastics under Simulated Sunlight Irradiation in Aquatic Environments: Roles of Reactive Oxygen Species. Water Res..

[113] Buchholz P.C.F., Feuerriegel G., Zhang H., Perez-Garcia P., Nover L.L., Chow J., Streit W.R., Pleiss J. (2022). Plastics Degradation by Hydrolytic Enzymes: The Plastics-Active Enzymes Database—PAZy. Proteins Struct. Funct. Bioinform..

[114] Hasan Anik A., Hossain S., Alam M., Binte Sultan M., Hasnine M.T., Rahman M.M. (2021). Microplastics Pollution: A Comprehensive Review on the Sources, Fates, Effects, and Potential Remediation. Environ. Nanotechnol. Monit. Manag..

[115] Rai P.K., Lee J., Brown R.J.C., Kim K.H. (2021). Micro- and Nano-Plastic Pollution: Behavior, Microbial Ecology, and Remediation Technologies. J. Clean. Prod..

[116] Gómez-Méndez L.D., Moreno-Bayona D.A., Poutou-Piñales R.A., Salcedo-Reyes J.C., Pedroza-Rodríguez A.M., Vargas A., Bogoya J.M. (2018). Biodeterioration of Plasma Pretreated LDPE Sheets by Pleurotus Ostreatus. PLoS ONE.

[117] Ho B.T., Roberts T.K., Lucas S. (2018). An Overview on Biodegradation of Polystyrene and Modified Polystyrene: The Microbial Approach. Crit. Rev. Biotechnol..

[118] Delacuvellerie A., Cyriaque V., Gobert S., Benali S., Wattiez R. (2019). The Plastisphere in Marine Ecosystem Hosts Potential Specific Microbial Degraders Including Alcanivorax Borkumensis as a Key Player for the Low-Density Polyethylene Degradation. J. Hazard. Mater..

[119] Auta H.S., Emenike C.U., Fauziah S.H. (2017). Screening of Bacillus Strains Isolated from Mangrove Ecosystems in Peninsular Malaysia for Microplastic Degradation. Environ. Pollut..

[120] Paço A., Duarte K., da Costa J.P., Santos P.S.M., Pereira R., Pereira M.E., Freitas A.C., Duarte A.C., Rocha-Santos T.A.P. (2017). Biodegradation of Polyethylene Microplastics by the Marine Fungus Zalerion Maritimum. Sci. Total Environ..

[121] Sánchez C. (2020). Fungal Potential for the Degradation of Petroleum-Based Polymers: An Overview of Macro- and Microplastics Biodegradation. Biotechnol. Adv..

[122] Jeyakumar D., Chirsteen J., Doble M. (2013). Synergistic Effects of Pretreatment and Blending on Fungi Mediated Biodegradation of Polypropylenes. Bioresour. Technol..

[123] Danso D., Chow J., Streita W.R. (2019). Plastics: Environmental and Biotechnological Perspectives on Microbial Degradation. Appl. Environ. Microbiol..

[124] Wei R., Oeser T., Schmidt J., Meier R., Barth M., Then J., Zimmermann W. (2016). Engineered Bacterial Polyester Hydrolases Efficiently Degrade Polyethylene Terephthalate Due to Relieved Product Inhibition. Biotechnol. Bioeng..

[125] Huang X., Cao L., Qin Z., Li S., Kong W., Liu Y. (2018). Tat-Independent Secretion of Polyethylene Terephthalate Hydrolase PETase in Bacillus Subtilis 168 Mediated by Its Native Signal Peptide. J. Agric. Food Chem..

[126] Moog D., Schmitt J., Senger J., Zarzycki J., Rexer K.H., Linne U., Erb T., Maier U.G. (2019). Using a Marine Microalga as a Chassis for Polyethylene Terephthalate (PET) Degradation. Microb. Cell Factories.

[127] Kumar M., Kumar M., Pandey A., Thakur I.S. (2019). Genomic Analysis of Carbon Dioxide Sequestering Bacterium for Exopolysaccharides Production. Sci. Rep..

[128] Kumar M., Thakur I.S. (2018). Municipal Secondary Sludge as Carbon Source for Production and Characterization of Biodiesel from Oleaginous Bacteria. Bioresour. Technol. Rep..

[129] (2021). EIA International Energy Outlook. https://www.eia.gov/outlooks/ieo/tables_side_xls.php.

[130] Rezania S., Oryani B., Park J., Hashemi B., Yadav K.K., Kwon E.E., Hur J., Cho J. (2019). Review on Transesterification of Non-Edible Sources for Biodiesel Production with a Focus on Economic Aspects, Fuel Properties and by-Product Applications. Energy Convers. Manag..

[131] Abomohra A.E.-F., Sheikh H.M.A., El-Naggar A.H., Wang Q. (2021). Microwave Vacuum Co-Pyrolysis of Waste Plastic and Seaweeds for Enhanced Crude Bio-Oil Recovery: Experimental and Feasibility Study towards Industrialization. Renew. Sustain. Energy Rev..

[132] Uzoejinwa B.B., He X., Wang S., Abomohra A.E.-F., Hu Y., He Z., Wang Q. (2020). Co-Pyrolysis of Seaweeds with Waste Plastics: Modeling and Simulation of Effects of Co-Pyrolysis Parameters on Yields, and Optimization Studies for Maximum Yield of Enhanced Biofuels. Energy Sources Part A Recover. Util. Environ. Eff..

[133] Uzoejinwa B.B., He X., Wang S., Abomohra A.E.-F., Hu Y., Wang Q. (2018). Co-Pyrolysis of Biomass and Waste Plastics as a Thermochemical Conversion Technology for High-Grade Biofuel Production: Recent Progress and Future Directions Elsewhere Worldwide. Energy Convers. Manag..

[134] Yuan C., Zhao S., Ni J., He Y., Cao B., Hu Y., Wang S., Qian L., Abomohra A. (2022). Integrated Route of Fast Hydrothermal Liquefaction of Microalgae and Sludge by Recycling the Waste Aqueous Phase for Microalgal Growth. Fuel.

[135] Almutairi A.W., Al-Hasawi Z.M., Abomohra A.E.F. (2021). Valorization of Lipidic Food Waste for Enhanced Biodiesel Recovery through Two-Step Conversion: A Novel Microalgae-Integrated Approach. Bioresour. Technol..

[136] Evangelisti S., Lettieri P., Borello D., Clift R. (2014). Life Cycle Assessment of Energy from Waste via Anaerobic Digestion: A UK Case Study. Waste Manag..

[137] Mahmud S., Haider A.S.M.R., Shahriar S.T., Salehin S., Hasan A.S.M.M., Johansson M.T. (2022). Bioethanol and Biodiesel Blended Fuels—Feasibility Analysis of Biofuel Feedstocks in Bangladesh. Energy Rep..

[138] Haosagul S., Oaew S., Prommeenate P., Sawasdee V., Pisutpaisal N. (2021). DNA Microarray for Detection and Identification of Sulfur Oxidizing Bacteria in Biogas Clean-up System. Energy Rep..

[139] Pugazhendhi A., Arvindnarayan S., Shobana S., Dharmaraja J., Vadivel M., Atabani A.E., Chang S.W., Nguyen D.D., Kumar G. (2020). Biodiesel from Scenedesmus Species: Engine Performance, Emission Characteristics, Corrosion Inhibition and Bioanalysis. Fuel.

[140] Shen W., Cao B., Mu M., Yuan C., Li C., Hu X., Wang S., Abomohra A. (2022). Monophenols Recovery by Catalytic Pyrolysis of Waste Sawdust over Activated Biochar from the Brown Macroalgae Hizikia Fusiformis: Mechanism and Life-Cycle Assessment. J. Anal. Appl. Pyrolysis.

[141] Oyebamiji O.O., Boeing W.J., Holguin F.O., Ilori O., Amund O. (2019). Green Microalgae Cultured in Textile Wastewater for Biomass Generation and Biodetoxification of Heavy Metals and Chromogenic Substances. Bioresour. Technol. Rep..

[142] Abomohra A.E.F., El-Sheekh M., Hanelt D. (2014). Pilot Cultivation of the Chlorophyte Microalga Scenedesmus Obliquus as a Promising Feedstock for Biofuel. Biomass Bioenergy.

[143] Hu N., Xu Y., Sun C., Zhu L., Sun S., Zhao Y., Hu C. (2021). Removal of Atrazine in Catalytic Degradation Solutions by Microalgae Chlorella Sp. and Evaluation of Toxicity of Degradation Products via Algal Growth and Photosynthetic Activity. Ecotoxicol. Environ. Saf..

[144] Shao W., Ebaid R., Abomohra A.E., Shahen M. (2018). Enhancement of Spirulina Biomass Production and Cadmium Biosorption Using Combined Static Magnetic Field. Bioresour. Technol..

[145] Soroosh H., Otterpohl R., Hanelt D. (2022). Influence of Hydraulic Retention Time on Municipal Wastewater Treatment Using Microalgae-Bacteria Flocs in Sequencing Batch Reactors. Bioresour. Technol. Rep..

[146] Soroosh H., Otterpohl R., Hanelt D. (2022). Influence of Supplementary Carbon on Reducing the Hydraulic Retention Time of Microalgae-Bacteria (Mab) Treatment of Municipal Wastewater. SSRN Electron. J..

[147] Admirasari R., Hindersin S., von Schwartzenberg K., Hanelt D. (2022). Nutritive Capability of Anaerobically Digested Black Water Increases Productivity of Tetradesmus Obliquus: Domestic Wastewater as an Alternative Nutrient Resource. Bioresour. Technol. Rep..

[148] Han S.F.S.-F., Jin W., Tu R., Abomohra A.E.-F.A.E.F., Wang Z.H.Z.-H. (2016). Optimization of Aeration for Biodiesel Production by Scenedesmus Obliquus Grown in Municipal Wastewater. Bioprocess Biosyst. Eng..

[149] Krishnamoorthy S., Manickam P., Muthukaruppan V. (2019). Evaluation of Distillery Wastewater Treatability in a Customized Photobioreactor Using Blue-Green Microalgae-Laboratory and Outdoor Study. J. Environ. Manag..

[150] Song C., Hu X., Liu Z., Li S., Kitamura Y. (2020). Combination of Brewery Wastewater Purification and CO2 Fixation with Potential Value-Added Ingredients Production via Different Microalgae Strains Cultivation. J. Clean. Prod..

[151] Leng L., Wei L., Xiong Q., Xu S., Li W., Lv S., Lu Q., Wan L., Wen Z., Zhou W. (2020). Use of Microalgae Based Technology for the Removal of Antibiotics from Wastewater: A Review. Chemosphere.

[152] Hemalatha M., Sravan J.S., Min B., Venkata Mohan S. (2019). Microalgae-Biorefinery with Cascading Resource Recovery Design Associated to Dairy Wastewater Treatment. Bioresour. Technol..

[153] Faisal S., Zaky A., Wang Q., Huang J., Abomohra A. (2022). Integrated Marine Biogas: A Promising Approach towards Sustainability. Fermentation.

[154] Abomohra A.E.-F., El-Naggar A.H., Alaswad S.O., Elsayed M., Li M., Li W. (2020). Enhancement of Biodiesel Yield from a Halophilic Green Microalga Isolated under Extreme Hypersaline Conditions through Stepwise Salinity Adaptation Strategy. Bioresour. Technol..

[155] Almutairi A.W. (2022). Full Utilization of Marine Microalgal Hydrothermal Liquefaction Liquid Products through a Closed-Loop Route: Towards Enhanced Bio-Oil Production and Zero-Waste Approach. 3 Biotech.

[156] Ashour M., Elshobary M.E., El-Shenody R., Kamil A.W., Abomohra A.E.F. (2019). Evaluation of a Native Oleaginous Marine Microalga Nannochloropsis Oceanica for Dual Use in Biodiesel Production and Aquaculture Feed. Biomass Bioenergy.

[157] Mishra A., Medhi K., Malaviya P., Thakur I.S. (2019). Omics Approaches for Microalgal Applications: Prospects and Challenges. Bioresour. Technol..

[158] Abomohra A.E.-F., Jin W., Tu R., Han S.-F., Eid M., Eladel H. (2016). Microalgal Biomass Production as a Sustainable Feedstock for Biodiesel: Current Status and Perspectives. Renew. Sustain. Energy Rev..

[159] Liu Y., Lu M., Zhang X., Sun Q., Liu R., Lian B. (2019). Shift of the Microbial Communities from Exposed Sandstone Rocks to Forest Soils during Pedogenesis. Int. Biodeterior. Biodegrad..

[160] Nolte T.M., Hartmann N.B., Kleijn J.M., Garnæs J., van de Meent D., Jan Hendriks A., Baun A. (2017). The Toxicity of Plastic Nanoparticles to Green Algae as Influenced by Surface Modification, Medium Hardness and Cellular Adsorption. Aquat. Toxicol..

[161] Lagarde F., Olivier O., Zanella M., Daniel P., Hiard S., Caruso A. (2016). Microplastic Interactions with Freshwater Microalgae: Hetero-Aggregation and Changes in Plastic Density Appear Strongly Dependent on Polymer Type. Environ. Pollut..

[162] Bhattacharya P., Lin S., Turner J.P., Ke P.C. (2010). Physical Adsorption of Charged Plastic Nanoparticles Affects Algal Photosynthesis. J. Phys. Chem. C.

[163] Sjollema S.B., Redondo-Hasselerharm P., Leslie H.A., Kraak M.H.S., Vethaak A.D. (2016). Do Plastic Particles Affect Microalgal Photosynthesis and Growth?. Aquat. Toxicol..

[164] Bellingeri A., Bergami E., Grassi G., Faleri C., Redondo-Hasselerharm P., Koelmans A.A., Corsi I. (2019). Combined Effects of Nanoplastics and Copper on the Freshwater Alga Raphidocelis Subcapitata. Aquat. Toxicol..

[165] Mao Y., Ai H., Chen Y., Zhang Z., Zeng P., Kang L., Li W., Gu W., He Q., Li H. (2018). Phytoplankton Response to Polystyrene Microplastics: Perspective from an Entire Growth Period. Chemosphere.

[166] Besseling E., Wang B., Lurling M., Koelmans A.A. (2014). Nanoplastic Affects Growth of *S. obliquus* and Reproduction of *D. magna*. Environ. Sci. Technol..

[167] Zhu H., Fu S., Zou H., Su Y., Zhang Y. (2021). Effects of Nanoplastics on Microalgae and Their Trophic Transfer along the Food Chain: Recent Advances and Perspectives. Environ. Sci. Process. Impacts.

[168] Huang R., Liu Z., Yan B., Li Y., Shi W. (2019). Layer-by-Layer Assembly of High Negatively Charged Polycarbonate Membranes with Robust Antifouling Property for Microalgae Harvesting. J. Membr. Sci..

[169] Feng L.J., Sun X.D., Zhu F.P., Feng Y., Duan J.L., Xiao F., Li X.Y., Shi Y., Wang Q., Sun J.W. (2020). Nanoplastics Promote Microcystin Synthesis and Release from Cyanobacterial Microcystis Aeruginosa. Environ. Sci. Technol..

[170] Li Z., Yi X., Zhou H., Chi T., Li W., Yang K. (2020). Combined Effect of Polystyrene Microplastics and Dibutyl Phthalate on the Microalgae Chlorella Pyrenoidosa. Environ. Pollut..

[171] Zhu Z.L., Wang S., Zhao F., Wang S., Liu F., Liu G. (2019). zhou Joint Toxicity of Microplastics with Triclosan to Marine Microalgae Skeletonema Costatum. Environ. Pollut..

[172] Liu G., Jiang R., You J., Muir D.C.G., Zeng E.Y. (2020). Microplastic Impacts on Microalgae Growth: Effects of Size and Humic Acid. Environ. Sci. Technol..

[173] Canniff P.M., Hoang T.C. (2018). Microplastic Ingestion by Daphnia Magna and Its Enhancement on Algal Growth. Sci. Total Environ..

[174] Seoane M., Gonzalez-Fernandez C., Soudant P., Huvet A., Esperanza M., Cid A., Paul-Pont I. (2019). Polystyrene Microbeads Modulate the Energy Metabolism of the Marine Diatom Chaetoceros Neogracile. Environ. Pollut..

[175] Zhao T., Tan L., Huang W., Wang J. (2019). The Interactions between Micro Polyvinyl Chloride (MPVC) and Marine Dinoflagellate Karenia Mikimotoi: The Inhibition of Growth, Chlorophyll and Photosynthetic Efficiency. Environ. Pollut..

[176] Chae Y., Kim D., An Y.J. (2019). Effects of Micro-Sized Polyethylene Spheres on the Marine Microalga Dunaliella Salina: Focusing on the Algal Cell to Plastic Particle Size Ratio. Aquat. Toxicol..

[177] Song C., Liu Z., Wang C., Li S., Kitamura Y. (2020). Different Interaction Performance between Microplastics and Microalgae: The Bio-Elimination Potential of Chlorella Sp. L38 and Phaeodactylum Tricornutum MASCC-0025. Sci. Total Environ..

[178] Ugya A.Y., Meguellati K., Aliyu A.D., Abba A., Musa M.A. (2022). Microplastic Stress Induce Bioresource Production and Response in Microalgae: A Concise Review. Environ. Pollut. Bioavailab..

[179] El-Sheekh M., Abomohra A.E. (2022). Handbook of Algal Biofuels: Aspects of Cultivation, Conversion, and Biorefinery.

[180] Marey R.S., Abo-Shady A.M., Khairy H.M., Abd El-Moneim A.M., Abomohra A. (2022). Enhanced Lipid Production and Essential ω-Fatty Acids Synthesis by the Hypersaline Biodiesel-Promising Microalga Tetraselmis Elliptica through Growth Medium Optimization. Biomass Convers. Biorefinery.

[181] Han S.-F., Jin W., Yang Q., El-Fatah Abomohra A., Zhou X., Tu R., Chen C., Xie G.-J., Wang Q. (2019). Application of Pulse Electric Field Pretreatment for Enhancing Lipid Extraction from Chlorella Pyrenoidosa Grown in Wastewater. Renew. Energy.

[182] Tu R., Jin W., Wang M., Han S., Abomohra A.E.-F., Wu W.-M. (2016). Improving of Lipid Productivity of the Biodiesel Promising Green Microalga Chlorella Pyrenoidosa via Low-Energy Ion Implantation. J. Appl. Phycol..

[183] de Farias Silva C.E., Bertucco A. (2016). Bioethanol from Microalgae and Cyanobacteria: A Review and Technological Outlook. Process. Biochem..

[184] Abomohra A.E.-F.A.E.-F., Elshobary M. (2019). Biodiesel, Bioethanol, and Biobutanol Production from Microalgae. Microalgae Biotechnology for Development of Biofuel and Wastewater Treatment.

[185] Wang S., Mukhambet Y., Esakkimuthu S., Abomohra A.E.-F. (2022). Integrated Microalgal Biorefinery–Routes, Energy, Economic and Environmental Perspectives. J. Clean. Prod..

[186] González-González L.M., Zhou L., Astals S., Thomas-Hall S.R., Eltanahy E., Pratt S., Jensen P.D., Schenk P.M. (2018). Biogas Production Coupled to Repeat Microalgae Cultivation Using a Closed Nutrient Loop. Bioresour. Technol..

[187] El-Dalatony M.M., Salama E.-S., Kurade M.B., Kim K.-Y., Govindwar S.P., Kim J.R., Kwon E.E., Min B., Jang M., Oh S.-E. (2019). Whole Conversion of Microalgal Biomass into Biofuels through Successive High-Throughput Fermentation. Chem. Eng. J..

[188] Faisal S., Naveed M., Kifayatullah S., Muhammad M. (2022). Plastic Recycling for Energy Production. Waste-to-Energy.

[189] Nanda S., Berruti F. (2021). Thermochemical Conversion of Plastic Waste to Fuels: A Review. Environ. Chem. Lett..

[190] Esakkimuthu S., Wang S., El A., Abomohra F., Esakkimuthu S., Wang S., Abomohra F. (2022). CO_2_-Mediated Energy Conversion and Recycling. Waste-to-Energy.

[191] Xu S., Elsayed M., Ismail G.A., Li C., Wang S., Abomohra A.E.F. (2019). Evaluation of Bioethanol and Biodiesel Production from Scenedesmus Obliquus Grown in Biodiesel Waste Glycerol: A Sequential Integrated Route for Enhanced Energy Recovery. Energy Convers. Manag..

[192] Tawfik A., Ismail S., Elsayed M., Qyyum M.A., Rehan M. (2022). Sustainable Microalgal Biomass Valorization to Bioenergy: Key Challenges and Future Perspectives. Chemosphere.

